# Effects of substrate conductivity on cell morphogenesis and proliferation using tailored, atomic layer deposition-grown ZnO thin films

**DOI:** 10.1038/srep09974

**Published:** 2015-04-21

**Authors:** Won Jin Choi, Jongjin Jung, Sujin Lee, Yoon Jang Chung, Cheol-Soo Yang, Young Kuk Lee, You-Seop Lee, Joung Kyu Park, Hyuk Wan Ko, Jeong-O Lee

**Affiliations:** 1 Advanced Materials Division, Korea Research Institute of Chemical Technology (KRICT), Daejeon, 305-343, South Korea; 2 Research Center for Convergence Nanotechnology, Korea Research Institute of Chemical Technology (KRICT), Daejeon, 305-343, South Korea; 3 College of Pharmacy, Dongguk University, Gyeonggido, 410-820, South Korea; 4 Eco-Solution Team, DMC R&D Center, Samsung Electronics, Suwon, 443-742, South Korea

## Abstract

We demonstrate that ZnO films grown by atomic layer deposition (ALD) can be employed as a substrate to explore the effects of electrical conductivity on cell adhesion, proliferation, and morphogenesis. ZnO substrates with precisely tunable electrical conductivity were fabricated on glass substrates using ALD deposition. The electrical conductivity of the film increased linearly with increasing duration of the ZnO deposition cycle (thickness), whereas other physical characteristics, such as surface energy and roughness, tended to saturate at a certain value. Differences in conductivity dramatically affected the behavior of SF295 glioblastoma cells grown on ZnO films, with high conductivity (thick) ZnO films causing growth arrest and producing SF295 cell morphologies distinct from those cultured on insulating substrates. Based on simple electrostatic calculations, we propose that cells grown on highly conductive substrates may strongly adhere to the substrate without focal-adhesion complex formation, owing to the enhanced electrostatic interaction between cells and the substrate. Thus, the inactivation of focal adhesions leads to cell proliferation arrest. Taken together, the work presented here confirms that substrates with high conductivity disturb the cell-substrate interaction, producing cascading effects on cellular morphogenesis and disrupting proliferation, and suggests that ALD-grown ZnO offers a single-variable method for uniquely tailoring conductivity.

Studies of various organic/inorganic structures and materials as cellular substrates are a current research priority, reflecting the fundamental importance of understanding cellular interfaces and their applications, which range from wound healing and bone and nerve regeneration to prosthetics and artificial tissues and organs. Cells are extremely sensitive to nano- or micron-sized natural/artificial surface topographies and chemistries, which may permanently change cell fate^1^,^2^,^3^,^4^,^5^,^6^,^7^. Depending on the cell type or application, different materials/topographies are required as cell substrates. For example, neuronal cells prefer conductive substrates, such as carbon nanotubes^8^, whereas bone tissue regeneration requires mechanically robust substrates^9^, and vascular implants favor fibrous supports^10^,^11^. Despite these general trends, a fundamental understanding of the mechanisms underlying such tendencies has remained elusive owing to the simultaneous contributions of multiple cell substrate parameters.

Electrically conductive substrates have recently been used as cell-stimulating interfaces, and the effects of electrical conductivity on cell behavior have been extensively investigated^12^,^13^,^14^,^15^. For example, Thrivikraman and colleagues investigated the cell behavior with hydroxyapatite (HA) and calcium titanate (CA) and concluded that cell proliferation was enhanced on more highly conducting CA^12^. Jun et al. showed that electrically conductive composite fibers of poly(L-lactide-co-ε-caprolactone) blended with polyaniline stimulate the differentiation of myoblast cells^13^. Baxter and colleagues showed that electrically active (polarized) hydroxyapatite exerts positive effects on bone cell growth^14^ and suggested that the adsorption of proteins and ions on the polarized substrate might be a possible mechanism. However, conductivity of the substrates investigated was too low (~10^−9^/Ohm·cm for CA) to draw meaningful conclusions. Maydanov et al. investigated the role of an electrically conductive cell substrate by growing astrocytes on Au, Pt, Si, or SiO_2_ substrates^15^. Pt substrates were found to promote astrocyte cell growth; the same metallic Au surfaces exerted the opposite effect. Although Au and Pt are metallic substrates, Si a semiconducting one, and SiO_2_ could be classified as an insulating substrate. Thus, the cell growth effects cannot be exclusively attributed to differences in electrical conductivity because these substrates possess chemically and physically diverse properties. These studies highlight the importance of being able to vary a single physical parameter while holding all other physicochemical parameters constant to develop a clear understanding of the effect of electrically conducting substrates on cell behavior.

In this work, we investigated ZnO films grown by atomic layer deposition (ALD) as cell-interfacing substrates with variable electrical conductivity. Depending on their thickness, ALD-grown ZnO films displayed a wide range of electrical properties, encompassing insulating, semiconducting and metallic properties, whereas their chemical and topological properties remained constant. SF295 glioblastoma cells grown on ZnO films with different conductivities exhibited marked differences in cell morphogenesis and proliferation that depended on the conductivity of the film.

## Results

### Preparation and characterizations of ZnO films

ZnO is a wide bandgap (3.37 eV at room temperature) group II-VI semiconductor material that is used in numerous fields of materials research^16^. Its optical clarity and relatively metallic properties allow it to be implemented as a transparent, conductive, oxide material for electrodes in smart windows and touch screens. In the semiconductor industry, ZnO is widely used as the active channel material in thin film transistors owing to its large on/off ratio and moderate field effective mobility, possibly even challenging classic Si-based devices in some applications^17^,^18^,^19^,^20^,^21^. ZnO is also commonly found as an optoelectronic film in various optical applications^22^, and its piezoelectric properties have opened a broad avenue of research in energy devices.

**Figure 1:**
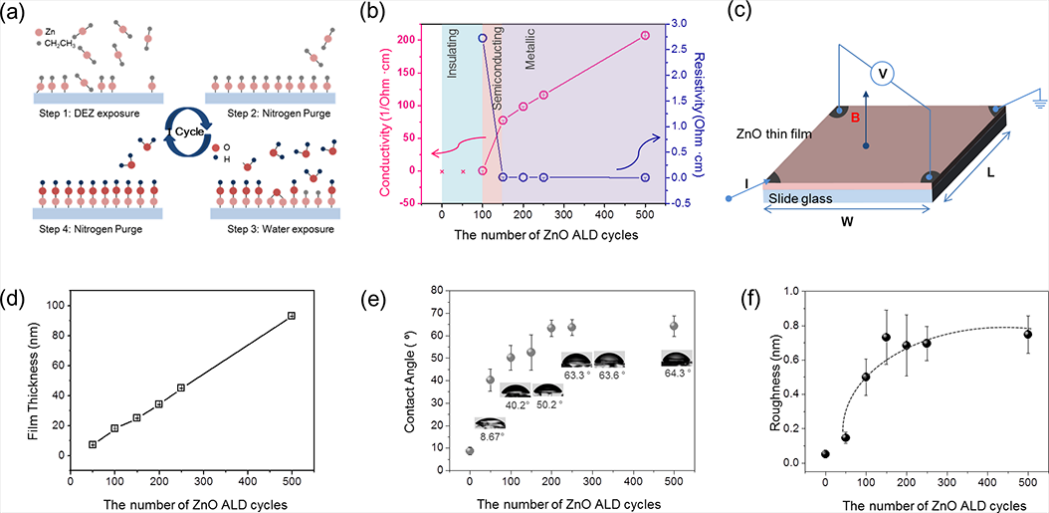
Characteristics of ALD-grown ZnO thin films. (a) A schematic depiction of the ALD process used to prepare ZnO thin films with different nanometer-depth thicknesses. (b) Conductivity versus ALD cycle number in ZnO thin films. ZnO films prepared with 50 and 100 ZnO-layering cycles are classified as insulators, those prepared with 150 cycles are classified as semi-conductors, and those prepared using more than 200 cycles are categorized as metal. (c) A scheme showing Hall measurements of ZnO thin films deposited on glass. (d) ZnO film thickness versus the number of ALD cycles. (e) The degree of hydrophobicity of ZnO thin films determined by measuring the contact angles. As the ZnO film becomes thicker, the contact angle increases from 40° to 64°. The contact angle becomes saturated after 200 cycles of ALD. (f) Plot of surface roughness, analyzed by AFM, versus the number of ALD cycles. The surface roughness curve exhibits a hyperbolic relationship with the cycle number (from 0.15 nm to 0.7 nm) and saturates at 150 cycles.

The ZnO thin films used here were grown on glass substrates using the ALD process shown in Figure 1a. A single cycle of ALD is composed of a pulse of diethyl zinc (DEZ) followed by a purge process, resulting in the formation of a layer of Zn-terminated bonds on the surface of the glass substrate. This cycle is then followed by a subsequent pulse of H_2_O to attach O atoms to these chains to form a layer (~0.2 nm) of ZnO^23^,^24^. The self-limited nature of ALD enables atomic-scale control of the thickness of ZnO films while maintaining other factors, such as surface roughness and chemical composition. The conductivity of ZnO films is generally governed by film thickness in the nanometer range, allowing the electrical properties of the film to be carefully tuned without altering other characteristics. Such tunable conductivity is unique to ALD-processed ZnO thin films, distinguishing this approach from other fabrication methods, such as sputtering, chemical vapor deposition, hydrothermal and sol-gel-derived ZnO.

Figure 1 (b–f) shows the properties of ALD ZnO thin films as a function of cycle number (50–500 cycles). A plot of the electrical characteristics of the ZnO thin films (Figure 1b), measured by the Hall effect (Figure 1c), shows that the very thin films (~7 nm; 50 cycles) are similar to insulators, exhibiting no measurable conduction. At intermediate thicknesses of ~18 nm (100 cycles) and ~25 nm (150 cycles), the films show semiconducting behavior with moderate conductivity (0.4–78 S/cm) and typical field effect transistor characteristics (Supplementary Figure S1). When the film thickness exceeds 34 nm (>200 cycles), the conductivity values are large enough to allow implementation as a metallic electrode (>100 S/cm). The conductivity values (Figure 1b) and charge carrier concentration values extracted/derived from them increased linearly with increases in the number of ALD cycles. Although the effect of substrate conductivity has been studied previously^12^, the present work employed the widest conductivity range, as well as much higher conductivity, highlighting the appropriateness of the current study. The correlation between the number of ALD cycles and film thickness is shown in Figure 1d. The thickness of the film was confirmed by atomic force microscopy (AFM).

Figure 1 (e and f) shows the hydrophobicity of the films determined from contact angle measurements and their roughness, measured by AFM. Both profiles clearly show that the surface properties, except charge carrier concentration, remained constant above ~200 cycles of ZnO thin film layering, implying that any changes in cell behavior beyond this point are unrelated to differences in the surface roughness or contact angle. Typical topographic images and wetting-angle measurements corresponding to a range of ALD cycles (50–500) are shown in Supplementary Figures S2 and S3.

### SF295 cell growth on ZnO thin films

**Figure 2:**
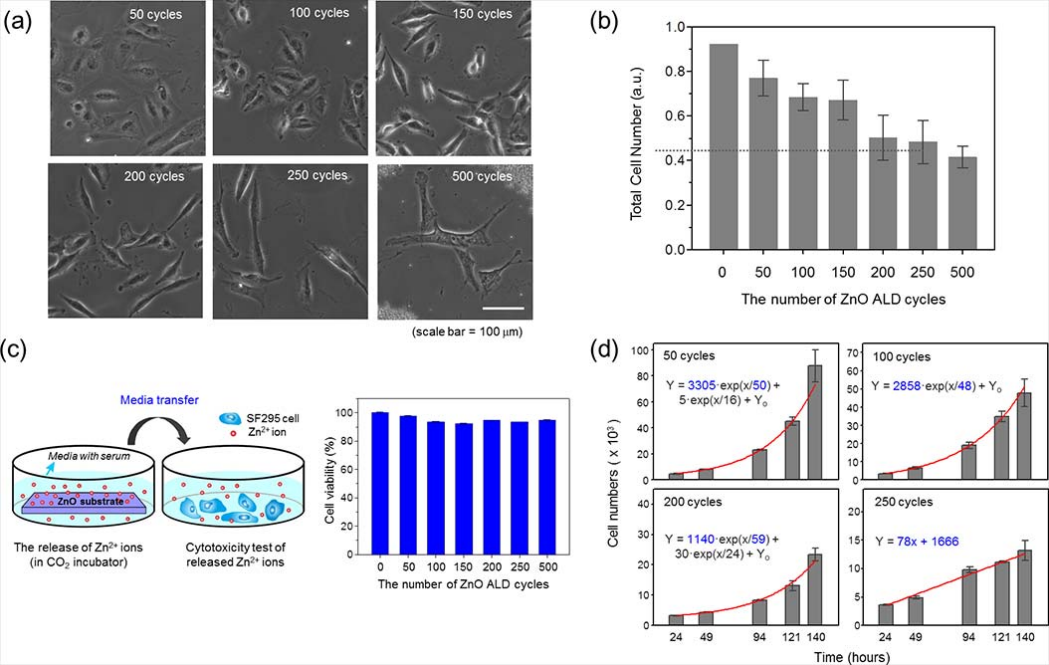
Cell viability and proliferation on ZnO films. (a) Differential-interference contrast (DIC) images of SF295 cells on ZnO thin films with different charge carrier densities (50–500 cycles of ZnO layering). SF295 cells on substrates with a higher carrier density exhibited more elongated and narrowed ends. Scale bar = 20 µm. (b) Histogram showing the optical density of SF295 cells grown on ZnO substrates expressed as absorbance units (a.u.) and determined using MTS assays. Assays were performed 7 days after seeding cells on each ZnO substrate. Data are presented as the means ± S.E.M. (n = 3). Significance was tested using one-way analysis of variance (ANOVA) with post hoc Fisher's least significant difference (LSD) analysis. *, P < 0.05; NS, not significant. (c) Possible contribution of the toxicity of released Zn^2+^ ions to the ALD cycle number-dependent effects of ZnO thin films on proliferation (shown in 2b). *Left:* Illustration of the experimental setup. ZnO thin films were incubated (or soaked in cell culture medium) for 24 hours in a CO_2_ incubator, after which the conditioned medium was collected. SF295 cells grown on glass slides were then incubated with the collected ZnO thin film-conditioned medium for 24 hours, and their viability was assayed by MTS assay. *Right:* Results of cell viability assays. The decrease in cell viability did not exceed 10% compared with controls incubated in conditioned medium from the glass substrate. Zn^2+^ ion release from ZnO thin films was further examined by measuring the concentration of Zn^2+^ ions using ICP-AES (see Supplementary Figure S7). Data are presented as the means ± S.D. (n = 3). Significance was tested using one-way ANOVA with post hoc Fisher's LSD analysis. ***, P < 0.001; NS, not significant. (d) Cell counting assay to quantify the proliferation of SF295 cells cultured on each ZnO substrate from 24 to 140 hours. Cell growth curves indicate that SF295 cells grown on glass or 50- to 200-cycle ZnO films proliferated exponentially, whereas cells grown on 250- and 500-cycle ZnO films showed linear growth.

To examine the effects of variable conductivity on cellular responses, the SF295 cell line was used as a model system. SF295 is an established high-grade brain tumor cell line derived from human malignant glioma^25^. Brain tumors are highly aggressive, yet the treatment therapy for brain tumors remains limited. Tumor treating fields (TTF) therapy was recently approved by the U.S. Food and Drug Administration (FDA) as a treatment method for this devastating malignant glioma^26^,^27^. TTF therapy uses low intensity alternating electric fields and currents, and the molecular basis of TTF presumably lies in the disruption of the mitotic process of proliferating cancer cells by alternating electric fields. In this regard, the interaction of glioma cells and the electric field (current) need to be explored at a basic level; thus, we explored cell motility and proliferation on substrates with varying electrical conductivity. SF295 cells were cultured on ZnO thin films with or without serum (see Materials and Methods). In serum-free medium, SF295 cells attached normally to ZnO substrates but failed to form proper membrane protrusions, such as filopodia and lamellipodia. In addition, the cells could not spread out and instead displayed round shapes (Supplementary Figure S4). In the presence of serum, SF295 cells spread out properly but exhibited different patterns of membrane protrusion that depended on the thickness of the ZnO film. As shown in Figure 2a and Supplementary Figure S5, cells grown on highly conductive ZnO films adopted a more elongated shape and were well spread out. As the number of ZnO cycles increased, the cell elongation factor values, measured as the long axis/short axis ratio, also increased. Supplementary Figure S6 shows actin filament structures in SF295 cells, revealing morphological changes in cells grown on ZnO films with different conductivities. Cells were stained using the F-actin-specific binding peptide, phalloidin, conjugated with an Alexa Fluor 488 fluorescent probe. On highly conductive ZnO thin films (>250 cycles), the cells formed actin stress fibers aligned along the long axis of the cells. A previous study showed that cells tend to align themselves along extracellular cues, such as mechanical, chemical and electrical stimulation^28^. In a recent review by Li et al., various examples of in vitro cell alignment techniques were summarized^28^. However, no external stimulation sources, such as electric field (current)^29^, unidirectional mechanical stress (stretch^30^, flow^31^, compression^32^ or topographic patterning^33^) or line-shaped chemical stimulation^34^, were applied to the system in this work; the directions of cell alignment were randomly oriented as shown in Figure 2 (a).

We also observed a decrease in the total number of cells with increases in conductivity, as shown in Figure 2a. To assess the viability of SF295 cells grown on ZnO thin films with different thicknesses (50–500 cycles), we performed MTS (3-[4,5-dimethylthiazol-2-yl]-2,5 diphenyl tetrazolium bromide) assays after culturing cells for 7 days. As shown in Figure 2b, the measured optical density (O.D.) decreased dramatically with increasing ZnO thickness; on 500-cycle ZnO thin films, the cell viability decreased to less than 50% of that in controls (cells cultured on glass slides). Although the physicochemical features of the cell substratum are critically important in determining cell adhesion and viability, our results indicate that decreases in total cell numbers might be attributable to the charge carrier concentration rather than other surface properties. The surface roughness and hydrophobicity of ZnO thin films increased linearly over a limited thickness window corresponding to 0 to 150 cycles, but both parameters saturated at approximately 150 cycles and remained constant (see Figure 1e and f).

Previous studies have suggested two primary possibilities to account for the cytotoxic properties of ZnO nanomaterials on cultured cells: i) the dissolution and release of toxic cations^35^,^36^ and ii) the production of reactive oxygen species (ROS)^37^,^38^. In the first case, a likely mechanism begins with the endocytosis of ZnO nanorods. In this scenario, the acidic environment generated inside endosomes leads to the ionization of ZnO into O^2−^ and free Zn^2+^ ions; the latter then causes cytotoxic effects on cells. These events could occur if the ZnO particle size is small enough to be confined within the cytoplasm of the cell. ROS-mediated cytotoxicity could occur if sample-handling procedures are not sufficiently stringent. For example, the exposure of ZnO films to UV light might result in generation of ROS that is due to oxygen defects in the films. The underlying molecular mechanism of ROS-dependent ZnO nanoparticle-induced cell death might be attributable to mitochondrial damage and the subsequent disruption of cellular respiratory functions^39^. However, such a scenario cannot be directly applied to our system that used a relatively smooth, continuous film-type ZnO as a stimulant.

To determine whether the Zn^2+^ ions released from thicker ZnO films mediate the cytotoxic effects on SF295 cells^40^,^41^, we performed the following control experiment. First, ZnO films of different thickness were incubated for 24 hours in growth medium, and the conditioned media containing released Zn^2+^ ions were collected. SF295 cells on a glass substrate were then cultured in the collected media for 48 hours, after which the percentage of viable cells was determined using MTS assays (Figure 2c). The amount of Zn^2+^ ions released was also determined by measuring the Zn^2+^ concentration in the conditioned medium using inductively coupled plasma atomic emission spectroscopy (ICP-AES). As shown in Supplementary Figure S7, the concentration of Zn^2+^ ions increased with increasing thickness of the ZnO films, and medium collected from cells grown on 500-cycle ZnO thin films contained the highest concentrations of Zn^2+^ ions. Despite the cycle number-dependent increase in Zn^2+^ concentration, there was less than a 10% change in the total number of cells at any cycle number (Figure 2c), indicating that the Zn^2+^ ions released from ZnO thin films did not significantly influence cell viability.

MTS assays not only provide information about cell viability, they also provide a measure of cell proliferation. Thus, a lower O.D. value in the MTS assay might indicate a reduced level of cell proliferation on ZnO thin films instead of poor cell viability. To clarify the effects of ZnO on cell fate, we quantified viable cells by fixing and counting cells at four different time points (from 24 to 140 hours) after culturing on ZnO thin films (Figure 2d). As shown in Supplementary Table 1 and Supplementary Figure S8, the proliferation rate of SF295 cells varied according to the conductivity of the ZnO films. SF295 cells grown on 250- and 500-cycle ZnO thin films exhibited a linear increase in proliferation, whereas cells grown on all other ZnO thin films grew exponentially. Because all other physical parameters (wetting angle, surface roughness) become saturated after approximately 150–200 cycles, these findings suggest that the three different electrical conductivity states of ZnO thin films—metallic, semiconducting and insulating—that depend on the charge carrier density, caused SF295 glioblastoma cells to proliferate at different rates. Therefore, we conclude that the electrical conductivity of the substrate is the dominating factor for cell proliferation above 200 cycles of ZnO (metallic conductivity range where other parameters, such as wettability and surface roughness of ZnO, saturate). However, other physical parameters may also aid cell proliferation when the conductivity ranges of the ZnO films lie in the insulating or semiconducting regions (below 200 cycles).

**Figure 3:**
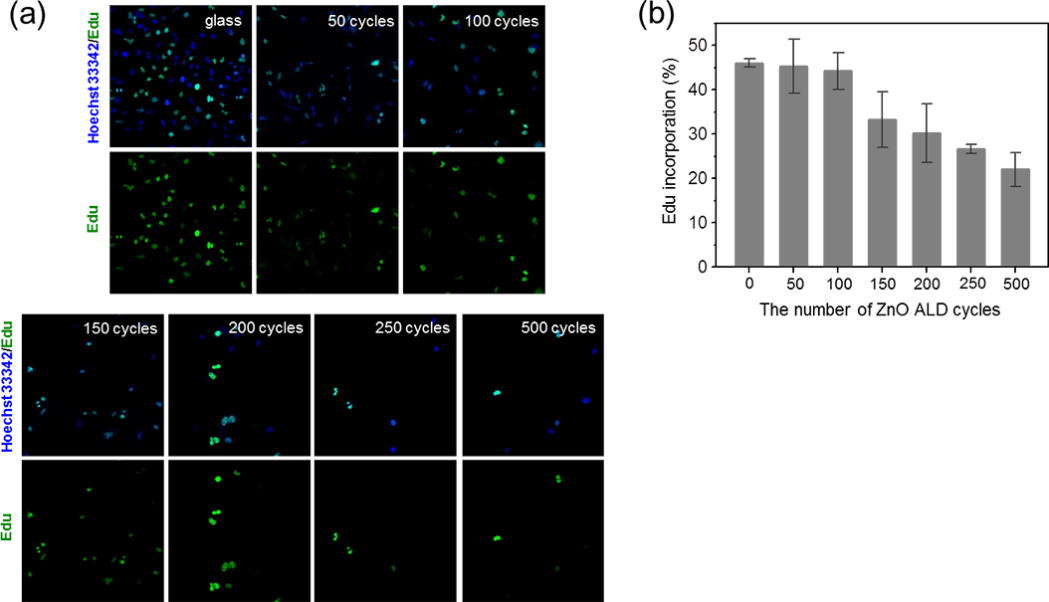
Fluorescence images of cell proliferation assayed using EdU. (a) Representative images of 1-hour pulse-labeled EdU staining (green) and nuclear staining (blue, Hoechst 3334). As the charge carrier density increased (high cycle numbers), the density of SF295 cells and percentage of proliferating cells decreased, as reflected by a decrease in the EdU-positive cell population. Scale bar = 20 μm. (b) Quantification of the ratio of EdU-incorporating cells to total cells. The percentage of proliferating SF295 cells on the 500-cycle ZnO substrate (28%) was less than that in controls cultured on a glass substrate (42%). Error bars denote standard errors of the mean (SEM; n = 4), and asterisks denote statistical significance based on one-way ANOVA and post hoc Fisher's LSD analysis. *, P < 0.05; NS, not significant.

We further examined the effects of changes in electrical conductivity on SF295 cell proliferation. To measure the degree of cell proliferation in SF295 cells, we used a labeling technique employing the non-radioactive thymidine analog, 5-ethynyl-2'-deoxyuridine (EdU), which is readily incorporated into the double-stranded DNA of proliferating cells. A single 1-hour pulse treatment of cells with EdU was followed by a 24-hour incubation, after which nascent DNA staining was analyzed using a copper-catalyzed reaction. This analysis revealed a significant decrease in the percentage of EdU-positive cells in ZnO films with a higher charge carrier density (Figure 3a and b). Cells grown on 500-cycle ZnO films showed an approximately 50% decrease in the EdU-positive fraction compared with control cells cultured on a glass plate. These results indicate that the electrical conductivity of the underlying substrate has an impact on the cell division rate. Then, what aspects of the electrically conductive substrate hinder cell proliferation? We found a clue from previous experimental work that showed that cell proliferation was reduced on adhesive substrates^42^.

### Adhesion force measurements of SF295 cells grown on ZnO thin films

**Figure 4:**
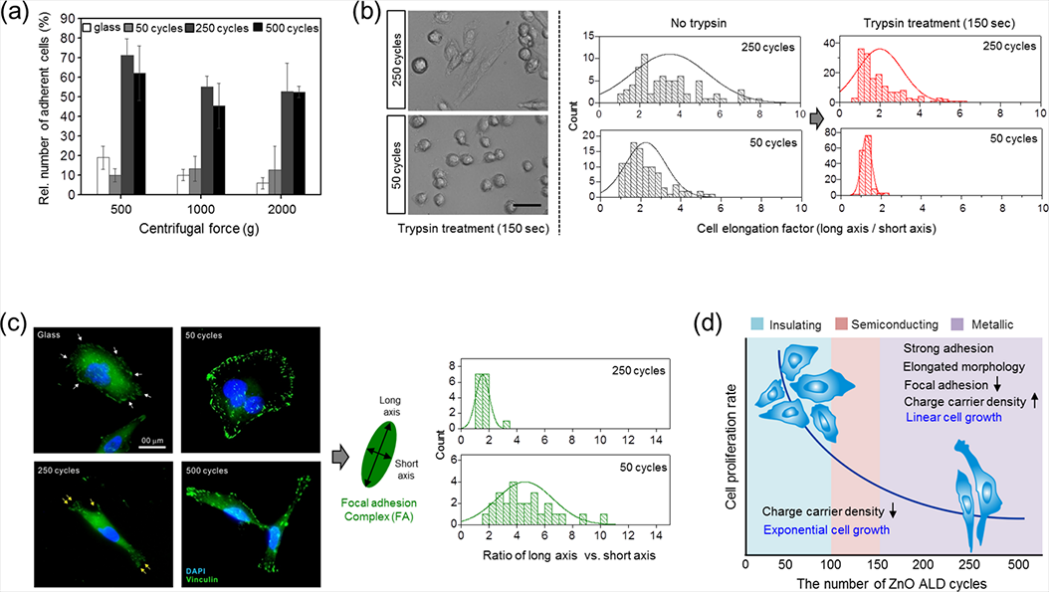
ZnO film thickness-dependent variations in charge carrier densities differentially affect SF295 cell adhesion and focal adhesion (FA) complex formation. (a) Quantitative assay of SF295 cell attachment assay on *insulating* substrates with a low charge carrier density (glass and 50-cycle ZnO films) compared with *metallic* substrates with a high charge carrier density (250- and 500-cycle ZnO films). Trypsin-treated SF295 cells, which adhered to each substrate, were centrifuged at different centrifugal forces (500, 1000, and 2000 × g), and then the cells were collected and counted (total cell number). More than three independent experiments were performed. Data are presented as the means ± SD (n = 3). Statistical significance was tested using one-way ANOVA with post hoc Fisher's LSD analysis. ***, P < 0.001; NS, not significant. (b) Changes in SF295 cell morphology after trypsin treatment, and quantification of the degree of cell adhesion on ZnO substrates. *Left:* DIC images. *Right:* Quantification, showing histograms of the cell elongation factor. Differences in cell elongation factor values reveal changes in cell morphology after trypsinization for 150 seconds (right histograms, red) compared with those before trypsinization (left histogram, gray). (c) FA complex analysis of SF295 cells grown on ZnO substrates. *Left panel:* Florescence images of vinculin staining with an Alexa 488-conjugated anti-vinculin antibody. Green rods and spots are vinculin-associated FA complexes, which are less abundant and aligned unidirectionally in cells grown on 250- and 500-cycle ZnO films. White arrows indicate conspicuous FA regions on each substrate. Higher magnification images of vinculin staining are shown in Supplementary Figure S10. *Right panel:* FAs were quantified by measuring the long axis length, short axis length, and long axis/short axis ratio. The distribution of FAs became more granular, and their expression level decreased as the charge carrier density increased to the metallic level (Supplementary Figures S10 and S11). (d) Summary of the different electro-conductive properties of ZnO thin films prepared with different ALD cycles and their effects on SF295 cell adhesion and proliferation.

The adhesion forces of cells grown on ZnO films with varying conductivity were analyzed as follows. First, cells growing on different substrates (varying conductivity) were treated with trypsin for 150 seconds and then centrifuged to remove weakly bound cells. The details of the adhesion force measurements are described in the Materials and Methods section. As shown in Figure 4a, the number of cells that remained attached to the substrate dramatically increased with increasing ZnO thickness. Figure 4b and Supplementary Figure S9, which compare cells grown on 50-cycle ZnO and 250-cycle ZnO after trypsin treatment for 150 seconds, clearly show that a larger number of cells grown on thicker ZnO films exhibit elongated cell shapes, indicating resistance to trypsin-EDTA or stronger adhesion.

How cells change their shape, biochemical characteristics, and motility in response to environmental cues has been extensively studied. Cells continuously perceive stimuli from the underlying surface and transduce these stimuli into specific intracellular signals to properly respond to changes in their surroundings. Specifically, integrin-based adhesion complexes are known to recognize the surrounding extracellular environment with extreme sensitivity; the biochemical properties, stiffness, and topography of the extracellular matrix (ECM) transduce signals into the interior of cells, causing a rearrangement of the cellular cytoskeleton^43^,^44^. Four different types of adhesion complexes are known to exist in fibroblasts: focal complexes, focal adhesions (FAs), fibrillar adhesions and three-dimensional matrix adhesions^43^. Of these complexes, the most important are FAs, which are large protein complexes composed of vinculin, talin, focal adhesion kinase (FAK), and paxillin. FAs are involved in many cellular events, including cell adhesion and migration. Several studies have shown that strong cell adhesion might occur through increases in the FA complex formation, which is highly correlated with the acceleration of cell proliferation. To explore the enhanced adhesion on conductive ZnO films, we examined the types of FA complexes in cells cultured on ZnO thin films by immunofluorescently staining cells with an anti-vinculin antibody. Immunofluorescence staining showed that the intensities and shapes of vinculin were different among cells growing on different ZnO thin film substrates. ZnO thin films with high conductivity caused weak and less locally concentrated vinculin staining, indicating decreased FA complex formation at intracellular surfaces (Figure 4c and Supplementary Figures S10 and S11). Consistent with this result, we also observed a decrease in immunofluorescence signals for tyrosine-phosphorylated FAK in SF295 cells seeded on 500-cycle ZnO thin films (Supplementary Figure 12). These results suggest that conductive ZnO substrates trigger the down-regulation of FA complexes containing vinculin and tyrosine-phosphorylated FAK. Moreover, whereas FAs with a highly elongated ellipsoid shape were most common in control cells grown on glass substrates, such FAs were less frequent in cells grown on ZnO thin films with high conductivity; these cells were more often round rather than elongated. A comparison of the size and shape of FAs, determined by measuring the length (long axis) and width (short axis) of individual vinculin-positive foci (Figure 4c), showed that the long axis/short axis ratio was smaller and more narrowly distributed in 250-cycle ZnO thin films than in 50-cycle ZnO films. These results indicate that conductive substrates with a higher charge carrier density prevent the growth of FA complexes in adhesive cells. When adhesive cells attach themselves on a substrate, focal complexes (nascent adhesins) are formed that initially have a rounded shape and a diameter of ~100 nm. On a conventional substrate, such focal complexes evolve into FAs, which are far more elongated and localized at the termini of stress fibers. However, focal complexes cannot mature into FAs on a conducting substrate and they remain as smaller and round focal complexes, as shown in Figure 4c.

## Discussions

**Figure 5:**
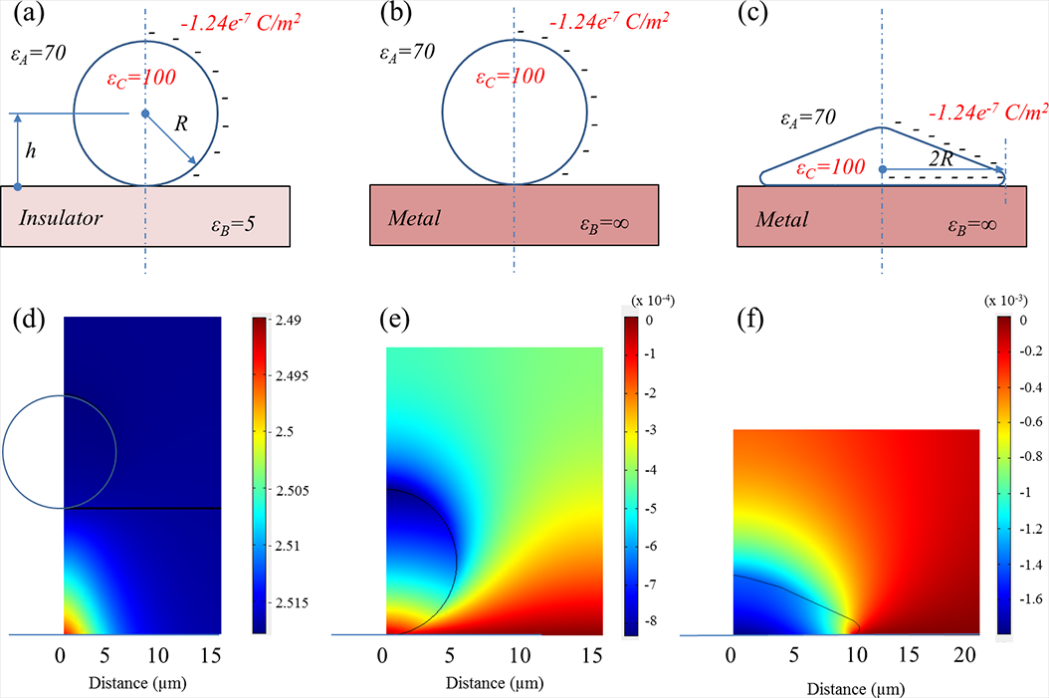
Schematic diagram showing the geometry used in modeling electrostatic forces exerted on a cell in contact with insulating (a) and metallic (b and c) substrates. In (c), the cell is deformed. The cell is modeled as a dielectric body (dielectric constant, 100) with a negatively charged surface (charge density, −1.24 × 10^−7^ C/m^2^) and a radius (R) of 5 μm. The cell is in medium with an assumed dielectric constant of 70. The dielectric constant of the insulating substrate is 5 and that of the metallic substrate is ∞. (d-f) Electric potential distribution map for the corresponding cases in the top row, simulated by the finite element method. The force on the dielectric body was calculated by integrating the electrostatic force on the dielectric surface over the electric field obtained from the electrostatic potential measurements. (a) When a cell (modeled as a dielectric sphere) is in contact with an insulating substrate with a dielectric constant of 5, the calculated force on the cell is 4.91 × 10^−15^ N (attractive). (b) When a cell is in contact with a metallic substrate, the force is 3.03 × 10^−15^ N (attractive). (c) When a cell in contact with a metallic substrate is deformed, as observed in the microscopic images, the calculated force is −1.01 × 10^−13^ N.

Conventional substrates, such as glass or plastics, are insulating, yet most cells display charges because of their membrane potential. Cells, which are considered charged spheres, may stick to insulating substrates through electrostatic interactions. By contrast, the counter charges developed by cells are not stable on metallic substrates; thus, cells are unable to stick to these surfaces and tend to slip away from them. Recently, Li et al. showed that a large-area graphene film transferred to a metallic substrate exhibited antibacterial activity compared with graphene transferred to insulating substrates^45^. Li and colleagues proposed that charges could be transferred from cells to a metallic (or semiconducting) substrate, thereby causing membrane damage. As a rough approximation, we modeled a cell as a dielectric sphere with a negative surface charge corresponding to a membrane potential of ~70 mV, a radius (R) of 5 μm and a dielectric constant of 100 and investigated the interaction of cells with metallic and insulating substrates. Figure 5 shows schematic diagrams of the cell interactions with insulating and metallic substrates and indicates the electric potential distribution between the cell and substrate calculated using a finite element method. In cells with a membrane potential of ~70 mV, the calculated charge density on the cell surface would be −1.24×10^−7^ C/m^2^. When such a cell is in contact with an insulating substrate with a dielectric constant of 5, the attractive force exerted on the cell is calculated to be 4.91×10^−15^ N, whereas that of cells in contact with a metallic substrate with dielectric constant of ∞ would be 3.03×10^−15^ N. Therefore, the adhesion force for cells on an insulating substrate is much larger than that on a metallic substrate. However, if the contact area between the cell and metallic substrate becomes larger through deformation of the cell, the adhesion force of the metallic substrate increases and becomes even larger than the values obtained for the insulating substrate. Because cells grown on a metallic substrate exhibit a more elongated shape (see Figure 1a), the cells may rearrange their cytoskeleton to adhere to the metallic substrate. Because of cytoskeleton deformation, the adhesion strength of deformed cells grown on a metallic substrate could be larger than that of cells grown on an insulating substrate. Additionally, as noted by Li and colleagues, cells may not activate the normal FA pathway, which involves the formation and subsequent maturation of the FA complex, owing to charge transfer from the cell membrane to the metallic substrate. Instead, cells deform their shapes to adhere to substrates with high conductivity; because of the disparity in adhesion mechanisms, cells adhered to a metallic substrate could not be easily removed in trypsin-based cell detachment assays.

In conclusion, we have demonstrated the potential of ALD-grown ZnO films as a model system for studying the effect of electrically conductive substrates on cell fate. The complete control of electrical conductivity, encompassing the full range of insulating to semiconducting and metal, was achieved with ALD, with minimal changes to other physico-chemical parameters of the ZnO films. Intriguingly, the SF295 glioblastoma cell line cultured on tailored ZnO films exhibited different behaviors depending on the conductivity of the film: cells on highly conductive ZnO displayed decreased proliferation and cytoskeletal rearrangements within the cell body that were clearly distinguishable from those of cells grown on a glass substrate (control) or ZnO substrates with lower conductivity. Moreover, using immunocytochemistry measurements of the adhesion complex and simple numerical calculation, we showed that it is difficult for cells to form FAs on conductive substrates, which translates into skeletal changes in the cells and the prevention of proliferation.

## Materials & Methods

### Deposition of ZnO thin films

The ZnO thin films were deposited by ALD in a Lucida D-100 chamber using diethylzinc (DEZ, electronic grade; Sigma-Aldrich, MO, USA) and H_2_O as the reactant and oxidant, respectively. All deposition schemes were performed under full saturation conditions, with DEZ-purge-H_2_O-purge cycles controlled at 0.5 seconds-10 seconds-0.1 second-30 seconds. The deposition temperature was fixed at 150°C. The canister temperature for both the source and oxidant was controlled by a Peltier device and was maintained at 15°C and 10°C for DEZ and H_2_O, respectively. This setting resulted in a peak pressure of ~1.5 torr during injection periods at a working pressure of ~1.36 torr.

### Fabrication and characterization of ZnO thin film field effect transistors

ZnO thin film transistors were fabricated using standard photolithographic processes in which an electrode of Al (100 nm) was deposited by thermal evaporation. The active channel in the device was defined by photolithographic patterning followed by etching with diluted nitric acid and had device dimensions of 40 μm (width) × 100 μm (length). Transport measurements of the devices were conducted under ambient conditions using a Keithley 4200-SCS semiconductor characterization system. Hall measurements were conducted using an HMS-3000 (Ecopia, Korea) in the Van der Pauw configuration, where the induced current was fixed to 1 mA to ensure proper electrical characterization for all cases. A Dimension 3100 atomic force microscope (Veeco, NY, USA) was employed to obtain topographical images and confirm the surface roughness.

### Cell culture on ZnO thin films

The culture of SF295 glioblastoma cells was performed according to a standard protocol, with slight modifications. Briefly, SF295 cells were cultured at 37°C in a humidified 5% CO_2_ atmosphere in RPMI-1640 containing 10% fetal bovine serum (FBS) and 1% streptomycin-penicillin (Invitrogen, CA, USA). Prior to seeding SF295 cells, ZnO thin film substrates (1.4 × 1.4 cm in 12-well plates) were sterilized in ethanol, washed with phosphate-buffered saline (PBS), and then pre-incubated in growth medium for 1–2 hours. To analyze cell morphology, SF295 cells grown on each substrate were fixed with 4% paraformaldehyde after 3–4 days of growth and then imaged using an Olympus IX81 inverted microscope system (Olympus, Japan). After culturing for 7 days, cells on each substrate were quantified using MTS assays as described by the manufacturer (Promega, CA, USA).

### Zn^2+^ ion cytotoxicity test

The cytotoxic effects of Zn^2+^ ions released from ZnO thin films were assessed by incubating two sets of each ZnO substrate in a 12-well plate in growth medium for 24 hours after sterilization. Conditioned medium from one set was used to determine the concentration of Zn^2+^ ions released from each ZnO substrate using duo inductively coupled plasma atomic emission spectroscopy (ICP-AES) (iCAP 6500; Thermo Scientific, Waltham, MA, USA). Conditioned medium from the second set was transferred to plates containing properly spread and growing SF295 cells. After 24 hours, cell viability under Zn^2+^ ion-rich conditions was assessed by MTS assay.

### Cell counting assay

SF295 cells grown on ZnO substrates were counted at specific times after seeding to estimate the effects of the depth of ZnO substrates on the cell proliferation rate. After seeding and culturing SF295 cells on each ZnO thin film, cells were fixed with 4% paraformaldehyde for 15 minutes and then stained with the fluorescent dye, Hoechst 33342, to facilitate cell nuclei counting. Cells were counted 24, 49, 94, 121, and 140 hours after seeding. Total cell populations at each time point were displayed as histograms, and differences in the cell proliferation rate as a function of the depth of ZnO thin films were determined by plotting cell growth curves.

### Cell proliferation assay

The proliferation rate of SF295 cells on ZnO films was determined using a Click-iT EdU Alexa Fluor 488 Imaging Kit (Molecular Probes, CA). SF295 cells were seeded on glass (control) or ZnO thin films. The next day, the cells were pulsed-labeled for 1 hour with EdU (10 μM). After replacing the cell growth medium with fresh medium, the label was chased by incubating cells for an additional 7–11 hours. After the chase period, the cells were fixed with 4% paraformaldehyde for 15 minutes and then stained with Click-iT reaction reagents as described by the manufacturer (Invitrogen, CA). In brief, fixed cells were rinsed twice with PBS/3% bovine serum albumin (BSA) and permeabilized with PBS/0.5% Triton X-100 for 20 minutes. The permeabilization buffer was replaced with 500 μL of Click-iT reaction cocktail to detect proliferating cells. The nuclei were stained with Hoechst 33342 following standard protocols. All staining procedures were performed at room temperature, and all fluorescence images were obtained using a Nikon C1 laser-scanning confocal microscope system equipped with a Nikon inverted fluorescence microscope (Nikon, Japan).

### Trypsin-treated cell adhesion assay

Cell adhesion on ZnO thin films was assessed by treating cells with trypsin followed by centrifugation. SF295 cells were cultured on glass (control), 50-cycle ZnO thin films (insulating), and 250- and 500-cycle ZnO thin films (metallic). After removing the culture medium and washing with PBS, a detachment rate with a gentle slope was achieved during centrifugation by treating each sample set with 0.01% trypsin-EDTA (diluted in PBS from a 0.05% trypsin-EDTA stock solution; Gibco, USA) at room temperature for 10–12 minutes. Culture medium was then added, and each sample was gently transferred to a 5 ml centrifugation tube (Eppendorf, Germany) and centrifuged in a swinging bucket rotor centrifuge (Allegra X-15R Centrifuge; Beckman Coulter Korea, Korea) at different relative centrifugal force (RCF) values (500, 1000, and 2000 × g) for 5 minutes. After centrifugation, each sample was transferred to a container containing PBS, and cells remaining on the substrates were detached by applying a strong fluid force using a micropipette. Centrifuged cells and forcibly detached cells were collected and counted using a hemocytometer.

The interfering effect of Zn^2+^ ions released from ZnO substrates on trypsin activity was assessed by treating SF295 cells on each substrate with the diluted trypsin solution under the same experimental conditions as described above for the cell adhesion experiments. After collecting and centrifuging the trypsin-treated solutions, the supernatants were added to SF295 cells in a multiwell plate. Changes in cell morphology were monitored, and the number of detached cells was compared. In addition, trypsin solutions collected from treated metallic substrates were applied to insulating substrates and vice versa. After incubation at room temperature for 10 minutes, detached cells and cells remaining on each substrate were collected and counted.

### Immunofluorescence staining of vinculin and FAK

For immunofluorescence labeling of cell adhesion components, SF295 cells were seeded on ZnO thin film substrates at the indicated densities. Cells were fixed with 4% (w/v) paraformaldehyde for 10 minutes, washed three times with PBS, and then permeabilized and blocked by incubating with PBS containing 1% horse serum and 0.1% Triton X-100 for 30 minutes. FA complexes were analyzed by incubating overnight at 4°C with anti-vinculin (1:400; Sigma) or phosphospecific anti-FAK [pY397] (1:1000; Invitrogen) antibodies. The next day, the cells were washed three times with blocking solution and then incubated with the appropriate fluorescein isothiocyanate (FITC)-conjugated secondary antibodies (1:2000, Jackson Immunoresearch) containing 4',6-diamidino-2-phenylindole (DAPI, 1:1000; Sigma) for 3 hours. After washing with blocking solution (PBS/1% horse serum), the samples were mounted on glass slides for subsequent imaging processing. All fluorescent images were acquired on a Nikon inverted fluorescence microscope (Nikon Instruments, Japan).

## Author Contributions

W.J.C. and J-O.L. designed and conducted the experiments employing ZnO thin films W.J.C. and Y.J.C. fabricated the substrates and analyzed the samples using AFM and contact angle and electrical transport measurements. J.J. and H.W.K. made plans for the cell experiments and characterization. J.J. determined the morphology, proliferation, and surface adhesion change in SF295 cells as the thickness of ZnO thin films was modulated. J.J. also performed the cell proliferation assay, cell counting assay, Zn^2+^ ion cytotoxicity test, quantification of released Zn^2+^ ions, and trypsin-treated centrifugation assay. S.L. performed the cell proliferation (EdU) assay, F-actin staining, vinculin and FAK staining experiments. C-S.Y. and Y-S.L. performed and analyzed the numerical calculations. W.J.C., J.J., S.L., Y.J.C, Y.K.L., H.W.K, and J-O.L prepared the figures and wrote the main manuscript text. J.K.P., H.W.K. and J-O.L. supervised the experimental aspects of the project. All authors contributed to the discussion and wrote the manuscript.

## Supplementary Material

Supplementary Information Supplementary informations
