# Supplementary material for: Effects of substrate conductivity on cell morphogenesis and proliferation using tailored, atomic layer deposition-grown ZnO thin films
Source: Sci Rep. 2015 Apr 21;5:9974. doi: 10.1038/srep09974 (PMC4404712; doi:10.1038/srep09974)

**Supplementary Information for**

**Effects of substrate conductivity on cell morphogenesis and proliferation using tailored, atomic layer deposition-grown ZnO thin films**

By *Won Jin Choi^1,†^, Jongjin Jung^2,†^, Sujin Lee^3,†^, Yoon Jang Chung^1^, Cheol-Soo Yang^1^, Young Kuk Lee^1^, You-Seop Lee^4^, Joung Kyu Park^2,*^, Hyuk Wan Ko^3,*^ and Jeong-O Lee^1,*^*

[1] W. J. Choi, Y. J. Chung, Dr. C. -S. Yang, Dr. Y. K. Lee, Dr. J. -O. Lee
Advanced Materials Division, Korea Research Institute of Chemical Technology (KRICT), Daejeon, 305-343, South Korea
E-mail: [jolee@krict.re.kr](mailto:jolee@krict.re.kr)

[2] Dr. J. J. Jung, Dr. J. K. Park
Research Center for Convergence Nanotechnology, Korea Research Institute of Chemical Technology (KRICT), Daejeon, 305-343, South Korea
E-mail: [parkjk@krict.re.kr](mailto:parkjk@krict.re.kr)

[3] S. Lee, Prof. H. W. Ko
College of Pharmacy, Dongguk University, Gyeonggido, 410-820, South Korea
E-mail: [kohw@dongguk.edu](mailto:kohw@dongguk.edu)

[4] Y. –S. Lee

Eco-Solution Team, DMC R&D Center, Samsung Electronics, Suwon, 443-742, South Korea

†These authors contributed equally to this work.

*Corresponding authors: Dr. Jeong-O Lee, Corresponding author e-mail: [jolee@krict.re.kr](mailto:jolee@krict.re.kr). Dr. Joung Kyu Park, Corresponding author e-mail: [parkjk@krict.re.kr](mailto:parkjk@krict.re.kr), Prof. Hyuk Wan Ko, Corresponding author e-mail: [kohw@dongguk.edu](mailto:kohw@dongguk.edu)

Keywords: Zinc oxide thin film, Charge carrier density, Conductivity, Glioblastoma, Cell adhesion, Proliferation

**Table of Contents**

|  |  |
| --- | --- |
| Figure S1. The physical characteristics of ZnO thin films as a semiconductor. |  |
| Figure S2. AFM images of ZnO thin films.  Figure S3. Contact angle measurements of ZnO thin films of different thickness. |  |
| Figure S4. DIC images of SF295 cells on ZnO thin films under serum-free conditions. |  |
| Figure S5. Quantification of changes in SF295 cell morphology on ZnO thin films. |  |
| Figure S6. Changes in SF295 cell morphology on ZnO thin films confirmed by staining for actin filament structures. |  |
| Figure S7. Determination of Zn^2+^ ions released from ZnO thin films. |  |
| Figure S8. Total cell population as a function of ZnO thin film cycle number and incubation time. |  |
| Table S1. Curve fitting formula and parameters for plots of cell proliferation histograms according to ZnO thin film cycle number and incubation time shown in Figure S5. |  |
| Figure S9. Tests of SF295 cell trypsin sensitivity before centrifugation according to the conductivity of substrates. |  |
| Figure S10. Immunofluorescence staining of vinculin (green), representing the density and directions of FAs in SF295 cells grown on insulating (glass and 50-cycle ZnO) and metallic (250- and 500-cycle ZnO) substrates. |  |
| Figure S11. Further analysis of FAs by immunofluorescence staining of vinculin. |  |
| Figure S12. Further analysis of FAs by immunofluorescence staining of FAK. |  |
| Figure S13. The number of FAs per cell determined by immunofluorescence staining of FAK. |  |
|  |  |


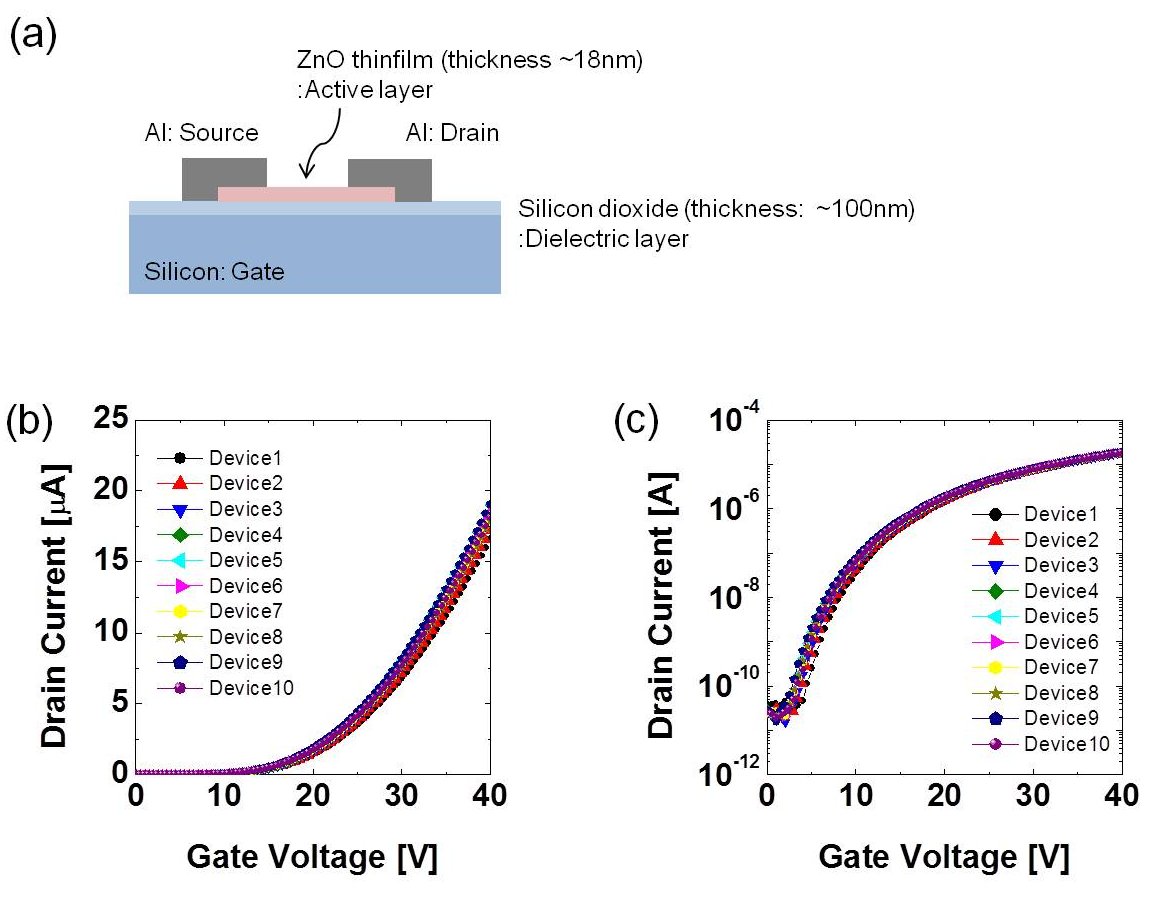


**Figure S1. The physical characteristics of ZnO thin films as a semiconductor.** (a) The schematic structure of a ZnO thin film (~18 nm, 100-cycle) field effect transistor. The ZnO thin film is deposited on a silicon dioxide/silicon substrate using the ALD method. Al is used as a source and drain electrode. (b) and (c) show the gate-modulated electrical characteristics in linear and log scale, respectively. These figures show that the ZnO thin film layer operates as a semiconductor.


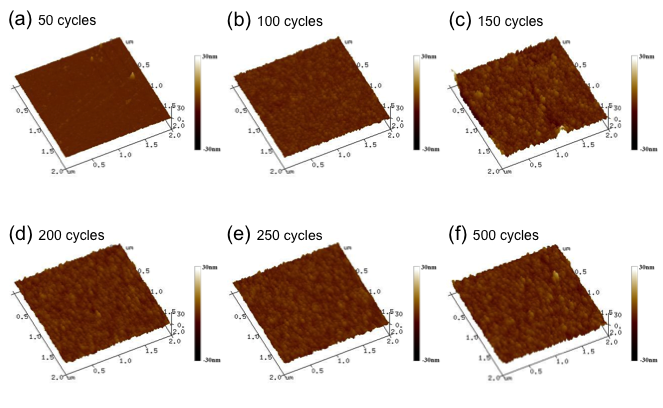


**S2.** **AFM images of ZnO thin films.** (a-f) AFM images of ZnO thin films with respect to the number of ALD cycles. Note comparable roughness of ZnO thin films at 150 ALD cycles and above.


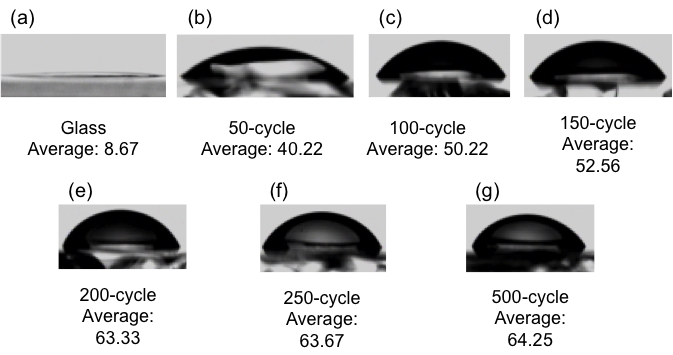


**Figure S3.** **Contact angle measurements of ZnO thin films of different thickness.**


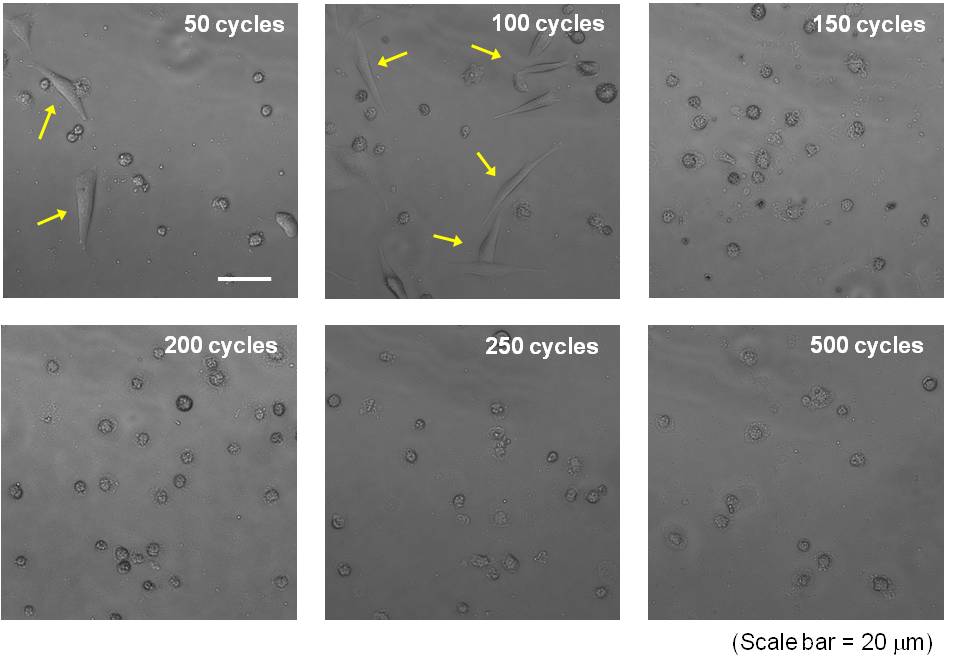


**Figure S4.** **DIC images of SF295 cells on ZnO thin films under serum-free conditions.** After sterilizing each ZnO substrate (50–500 cycles, 1.4 × 1.4 cm in 12-well plates) in ethanol and washing with PBS, SF295 cells in serum-free RPMI media were seeded. After 3–4 days, cells were fixed with 4% paraformaldehyde and imaged using an Olympus IX81 inverted microscope. In serum-free media, most SF295 cells attached to ZnO substrates, but only a few attached cells grown on insulators (50 and 100 cycles) formed proper membrane protrusions (filopodia and lamellipodia), and instead adopted a round shape. Unlike serum-containing conditions, all cells grown on semiconductors and metals (150–500 cycles) in the absence of serum maintained round and granular shapes and did not form any membrane protrusions.


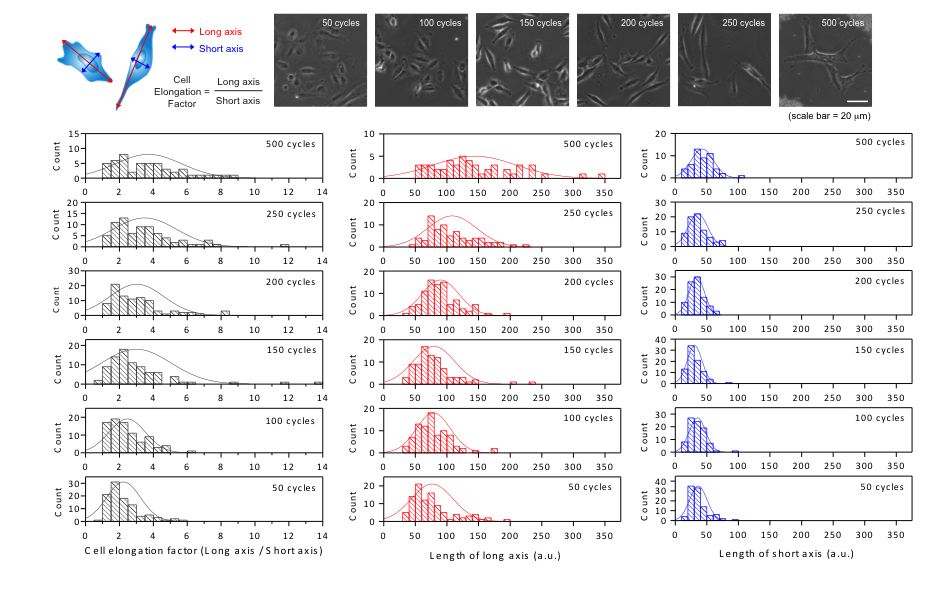


**Figure S5.** **Quantification of changes in SF295 cell morphology on ZnO thin films.** DIC images of SF295 cells on ZnO thin films (Fig. 2a) were further analyzed by measuring cell dimensions. Changes in cell morphology were quantified based on the cell elongation factor, defined as the ratio of the long axis to the short axis of a cell (top left). The distributions of measured values of cell elongation factor (gray), long axis length (red), and short axis length (blue) are represented as histograms (bottom). As the ALD-ZnO cycle number increased, the long axis elongated and the cell elongation factor value increased.


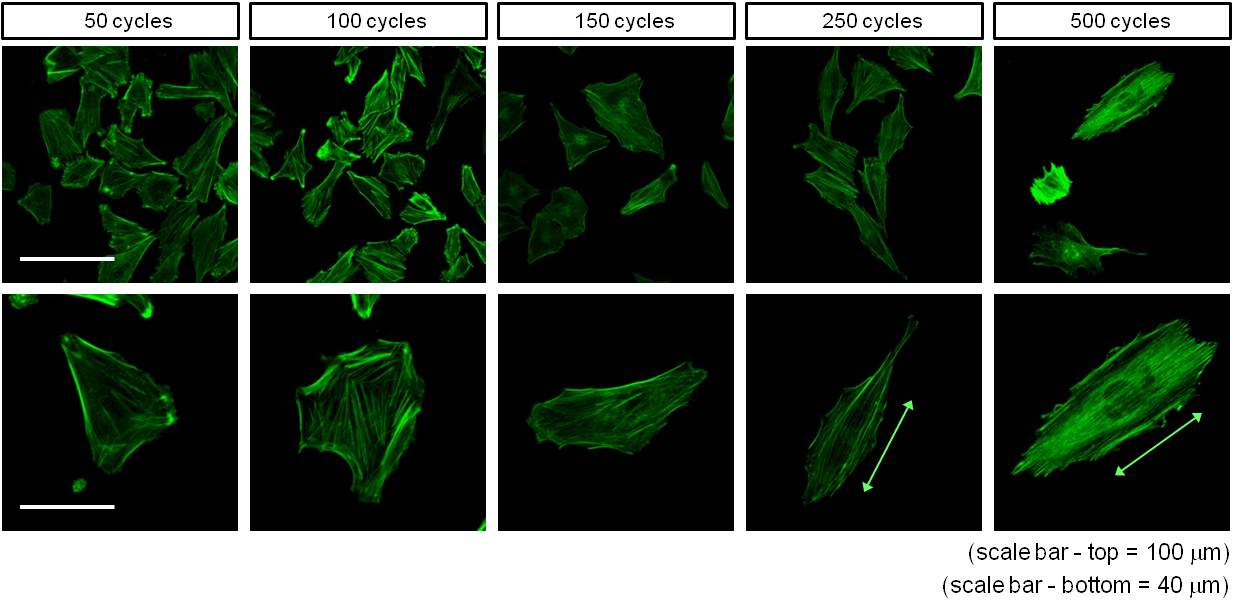


**Figure S6.** **Change in SF295 cell morphology on ZnO thin films affirmed by actin filament structures.** Fluorescence images of SF295 cell actin filaments stained with Alexa 488-conjugated phalloidin. The morphology and density of SF295 cells changed with increasing cycles of ZnO thin films. Arrows on images of 250- and 500-cycle ZnO substrates indicate the direction of actin stress fiber formation in SF295 cells. Scale bar = 100 μm (top) and 40 μm (bottom).


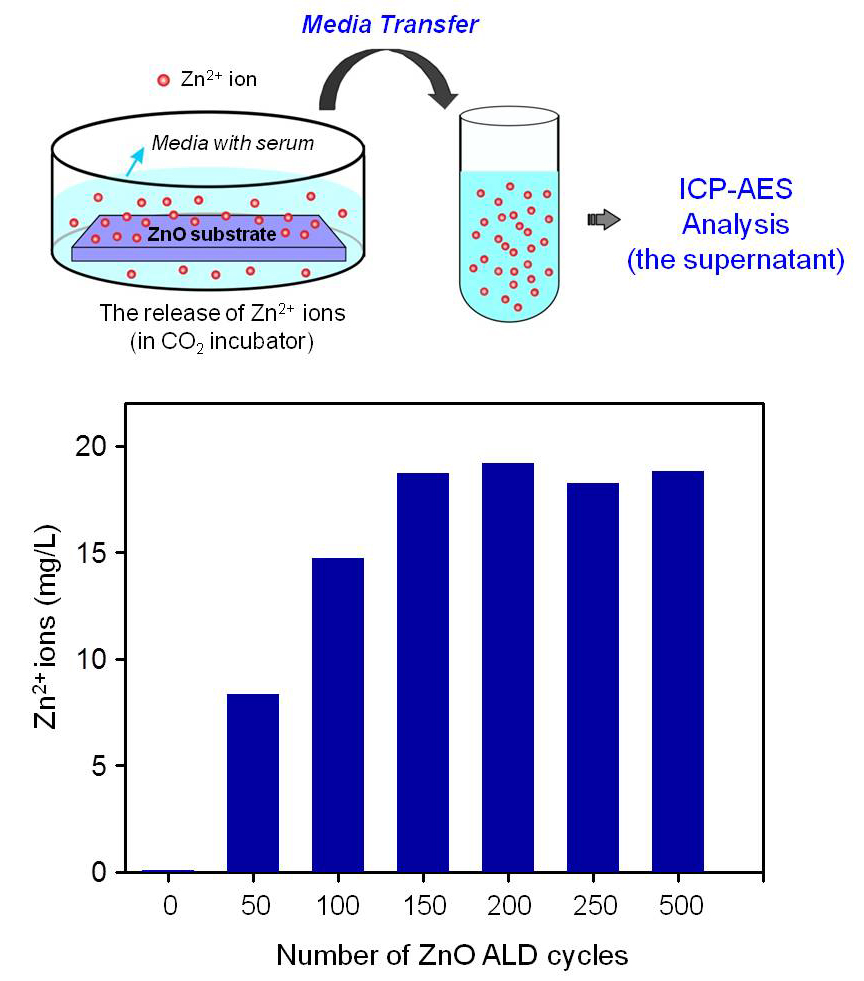


**Figure S7.** **Determination of Zn^2+^ ions released from ZnO thin films.** As part of the quantitative assessment of the cytotoxic effects of Zn^2+^ ions released from ZnO substrates, the concentration of Zn^2+^ ions was determined in ZnO thin film-conditioned media using ICP-AES. *Top:* Schematic depiction of the procedure. ZnO thin films were soaked in growth media for 24 hours at 37°C in a humidified 5% CO_2_ atmosphere, and the supernatant from each ZnO substrate was collected for ICP-AES analysis. *Bottom:* Concentration of Zn^2+^ ions according to the ZnO thin film cycle number. The concentration of Zn^2+^ ions in conditioned media was approximately 20 mg/L for ZnO substrates with cycle numbers ≥ 150. All experiment conditions were the same as those used for Zn^2+^ ion cytotoxicity tests.


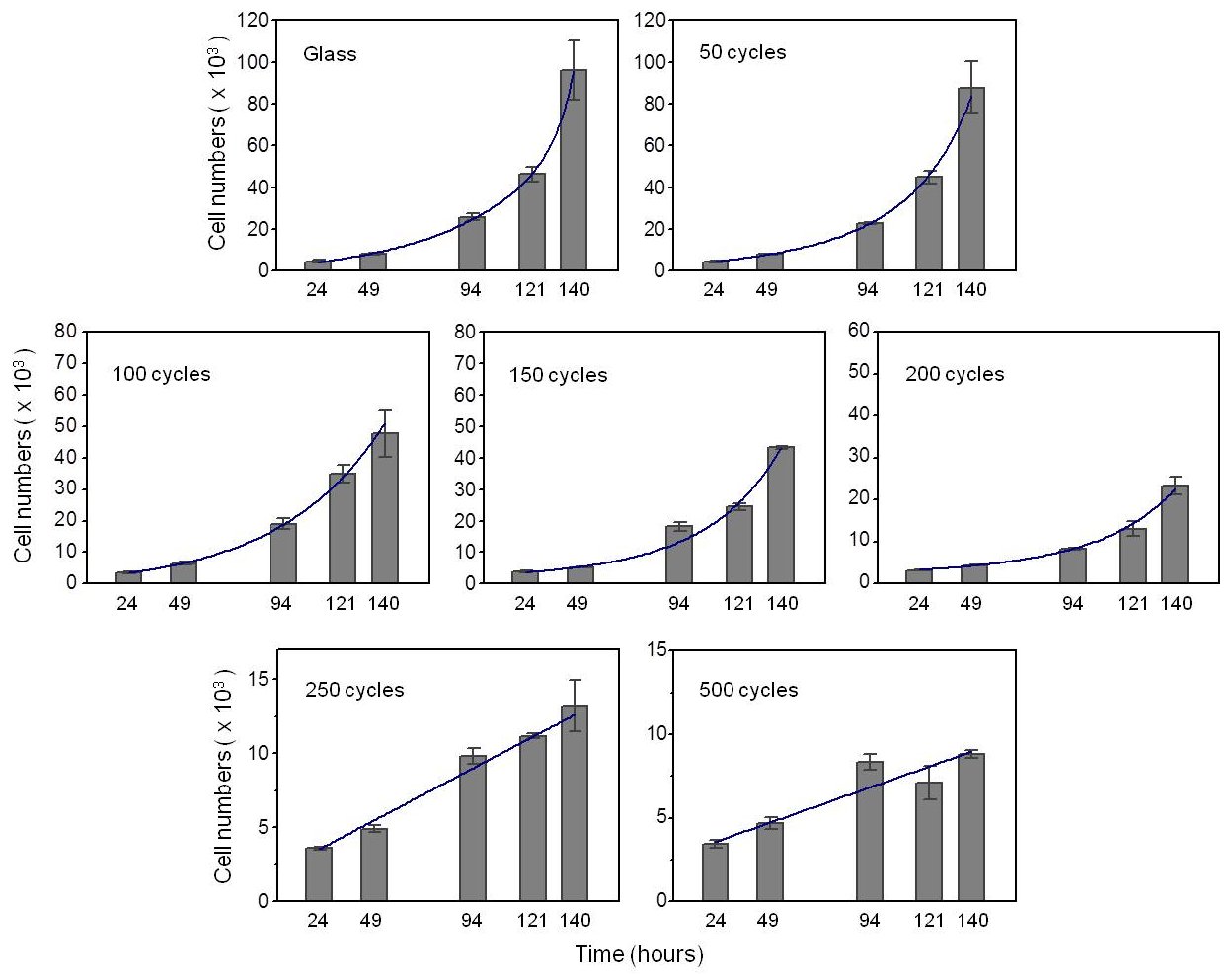


**Figure S8.** **Total cell population as a function of ZnO thin film cycle number and incubation time.** Compared to the cell proliferation rate on the bare glass substrate, cell growth rates progressively decreased as the ZnO thin film cycle number increased. Interestingly, SF295 cells on insulators and semiconductors (50–200 cycles) proliferated exponentially, whereas cells grown on substrates with metallic properties (250–500 cycles) grew linearly.

**Table S1.** Curve fitting formula and parameters for plots of cell proliferation histograms according to ZnO thin film cycle number and incubation time, shown in Figure S5**.**


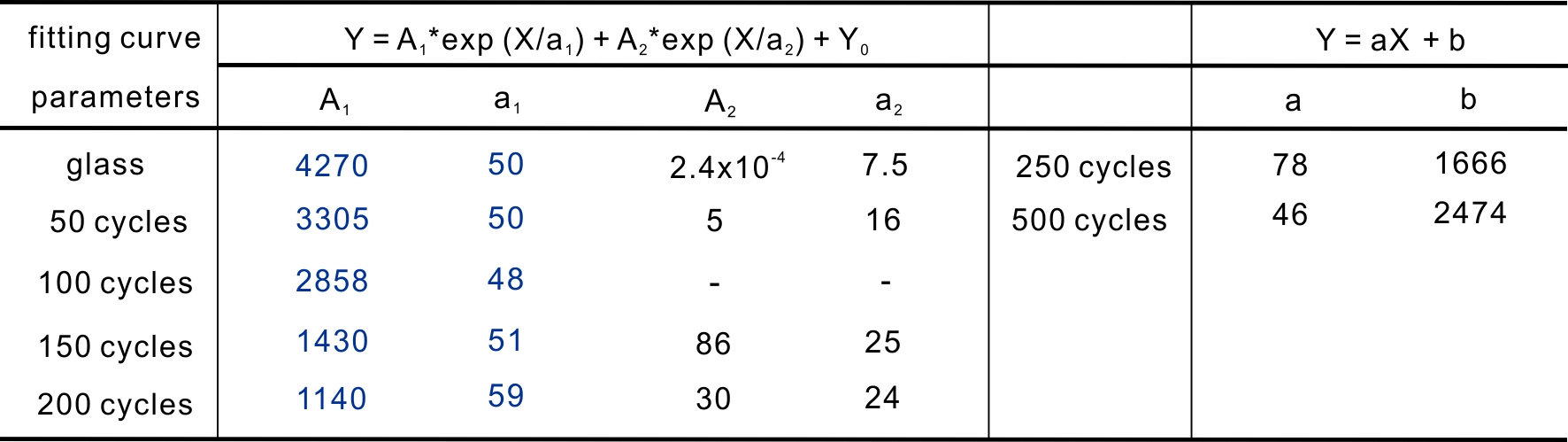


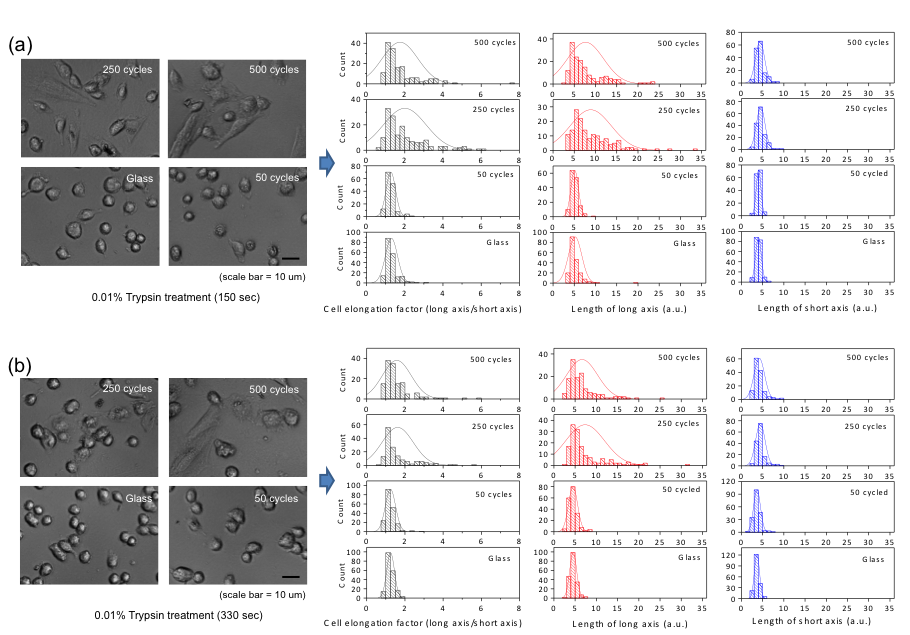


**Figure S9.** **Tests of SF295 cell trypsin sensitivity before centrifugation according to substrate conductivity.** SF295 cells cultured on insulating (glass and 50-cycle ZnO) and metallic (250- and 500-cycle ZnO) substrates were treated with a 0.01% trypsin-EDTA solution at room temperature for 150 seconds (a) and 330 seconds (b). *Left:* DIC images of cells fixed with 4% paraformaldehyde after trypsin treatment. Scale bar = 10 μm. *Right:* Histograms representing the long axis length (red), short axis length (blue), and the long axis/short axis ratio (gray) for SF295 cells shown on the left and in other DIC images. SF295 cells on insulating substrates were sensitive to trypsin protease activity to the extent that most cells shrunk and formed granular shapes after treatment for 330 seconds. However, many cells on metallic ZnO substrates showed resistance to trypsin activity, showing slowed shrinkage and retention of original morphologies. As incubation time increased from 150 to 330 seconds, the population of elongated cells decreased slightly, as evidenced by values for cell elongation factor and long axis lengths for SF295 cells on 250- and 500-cycle ZnO shown in the histograms on the right.


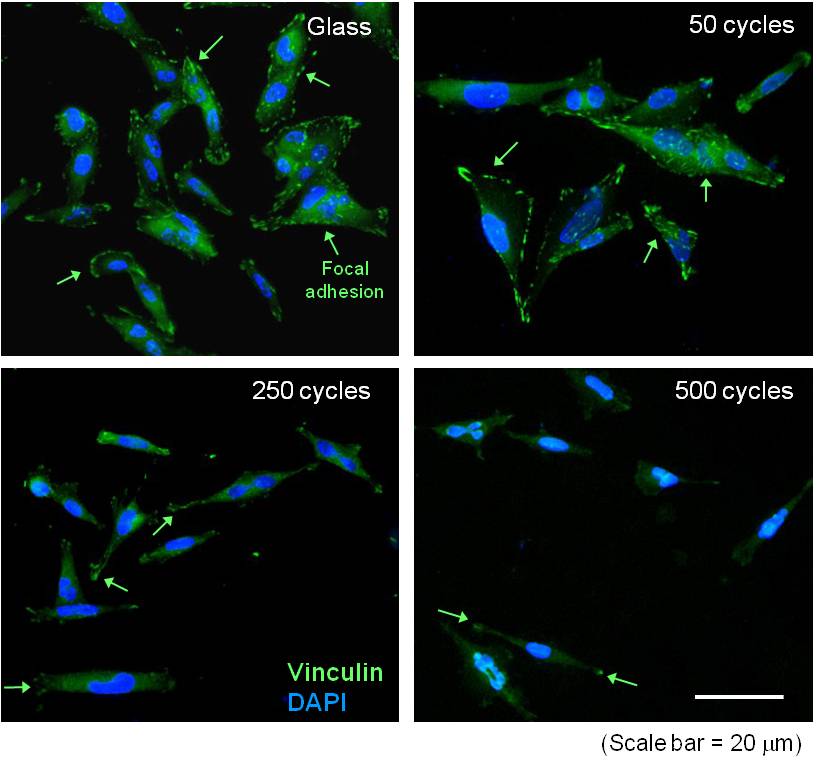


**Figure S10.** **Immunofluorescence staining of vinculin (green), representing the density and directions of FAs in SF295 cells grown on insulating (glass and 50-cycle ZnO) and metallic (250- and 500-cycle ZnO) substrates.** All images were collected using the same illuminating light intensity and exposure time in order to estimate the expression level of vinculin and FAs. On insulating substrates (glass and 50-cycle ZnO), FAs formed well and were distributed in various directions. However, on metallic substrates (250- and 500-cycle ZnO), FAs were limited in terms of both expression level and direction, with most FAs localized to the end of the cell body and aligned parallel with the long axis of the cell body. The nuclei were labeled with DAPI (blue).


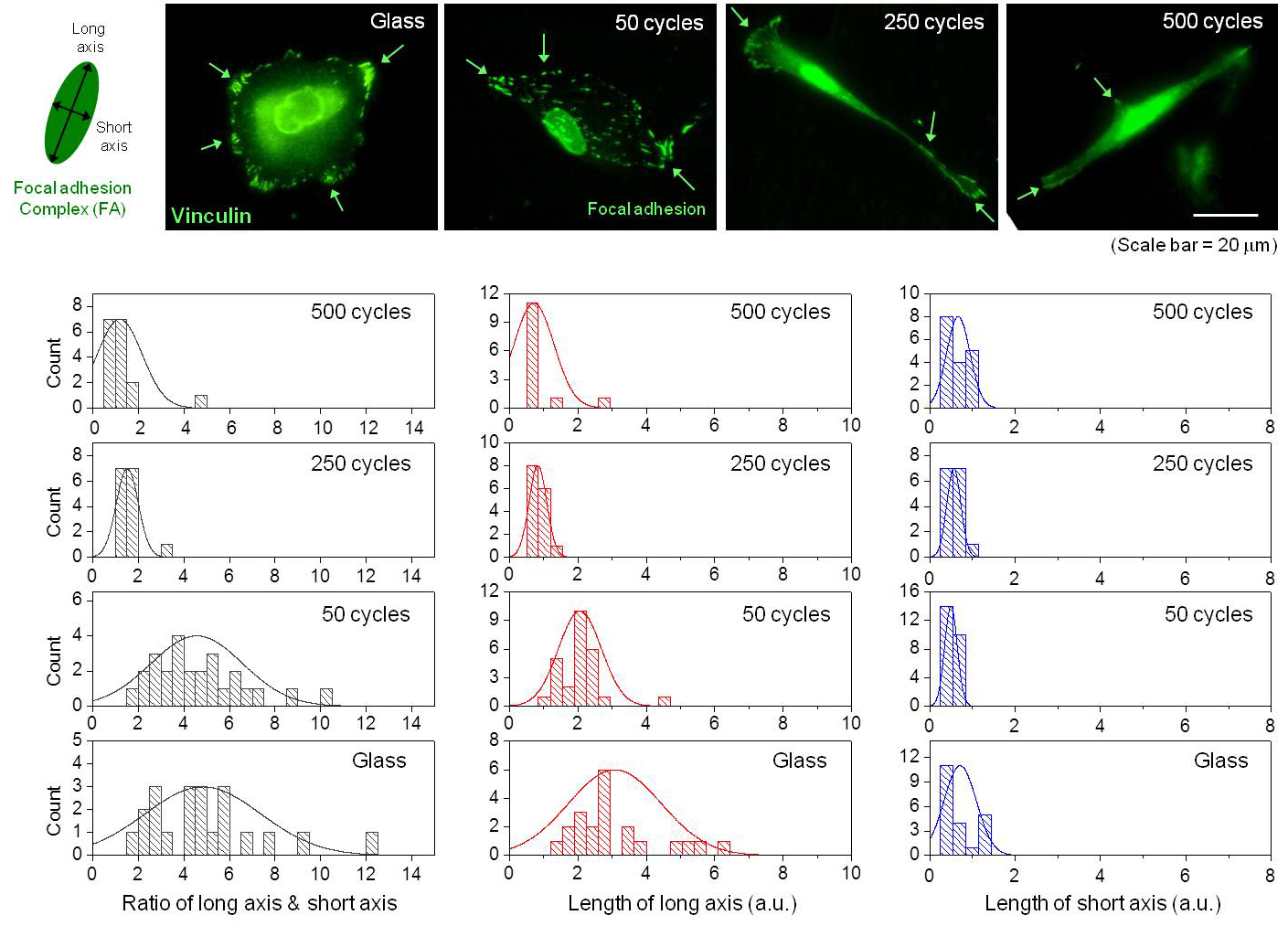


**Figure S11. Further analysis of FAs by immunofluorescence staining of vinculin.** *Top:* Fluorescence images show FAs in SF295 cells on insulating (glass and 50-cycle ZnO) and metallic (250- and 500-cycle ZnO) substrates. Rod or dot-like shapes with strong green staining represent vinculin in FA complexes. *Lower section:* The dimensions of FAs imaged by vinculin staining were quantified by measuring the long axis (red), short axis (blue), and the long axis/short axis ratio (gray). On insulating substrates, cells spread out properly and FAs were aligned in various directions, but on metallic substrates, cells were more elongated and narrow, and FAs formed mainly at the end regions of the long axis. FAs were predominantly rod-type in SF295 cells on insulating substrates. In contrast, dot-type FAs formed in cells on metallic substrates.


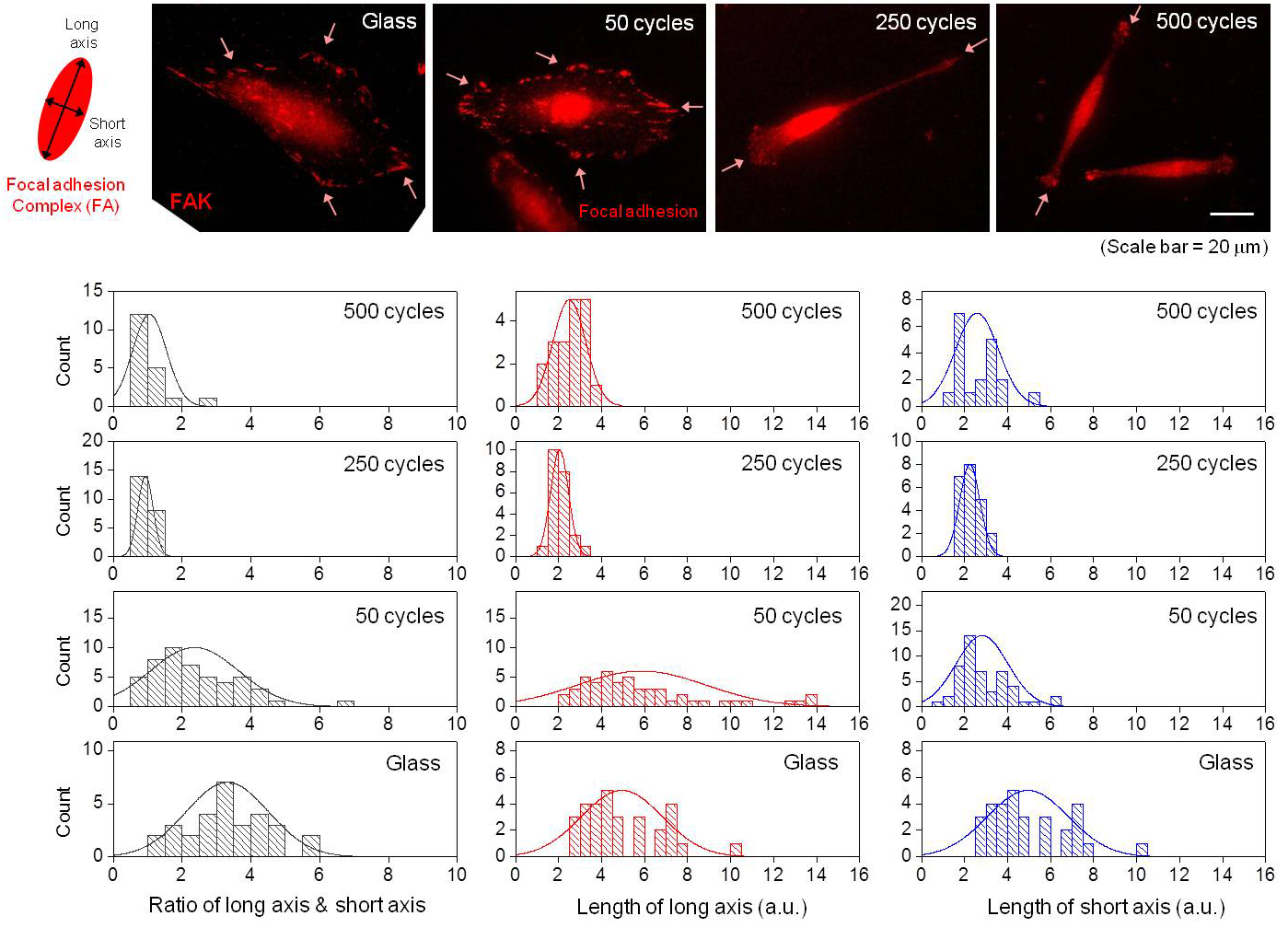


**Figure S12. Further analysis of FAs by immunofluorescence staining of FAK.** *Top:* Fluorescence images show FAs in SF295 cells on insulating (glass and 50-cycle ZnO) and metallic (250- and 500-cycled ZnO) substrates. Rod and dot-like shapes with strong red staining represent FAKs in FA complexes. *Lower section:* The dimensions of FAs imaged by FAK staining were quantified by measuring the long axis (red), short axis (blue), and long axis/short axis ratio (gray). On insulating substrates, cells spread out properly and FAs were aligned in various directions, but on metallic substrates, cells were more elongated and narrow and FAs formed mainly at the end regions of the long axis. FAs were predominantly rod-type in SF295 cells on insulating substrates. In contrast, dot-type FAs formed in cells on metallic substrates.


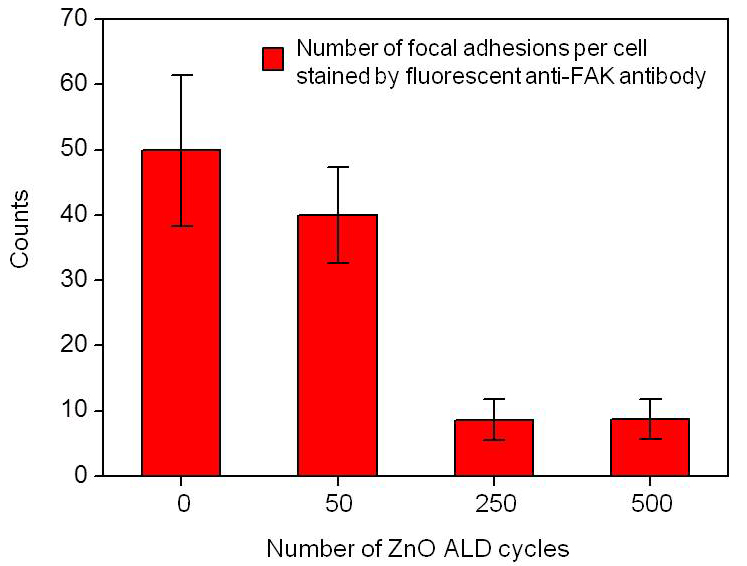


**Figure S13.** **The number of FAs per cell determined by immunofluorescence staining of FAKs.** FAs shown in Figure S11 were counted and averaged to estimate the dependence of FA formation on substrate properties. The number of FAs in SF295 cells on metallic substrates decreased and their dimensions changed from rod to dot type.


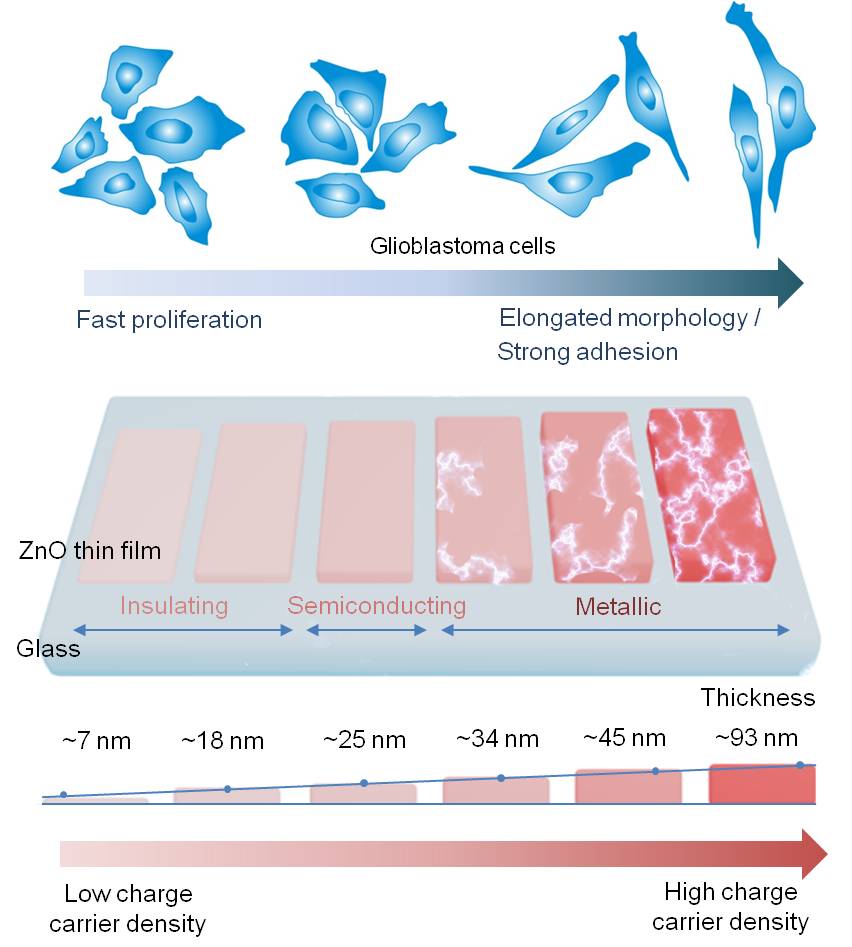

Supplement: Supplementary Information — s [file srep09974-s1.doc]
